# Brief communication: gaps and opportunities in HIV research in Venezuela

**DOI:** 10.1186/s12981-025-00700-4

**Published:** 2025-01-12

**Authors:** Jesús A. Morgado, María G. Medina, Rafael N. Guevara, Martín Carballo, Jaime R. Torres, Fhabián S. Carrión-Nessi, David A. Forero-Peña

**Affiliations:** 1 Biomedical Research and Therapeutic Vaccines Institute, Ciudad Bolívar, Venezuela; 2 “Luis Razetti” School of Medicine, Universidad Central de Venezuela, Caracas, Venezuela; 3 Department of Infectious Diseases, Hospital Universitario de Caracas, Caracas, Venezuela; 4 Instituto de Medicina Tropical “Dr. Félix Pifano”, Universidad Central de Venezuela, Caracas, Venezuela

**Keywords:** HIV, Systematic review, Venezuela, Research Gaps

## Abstract

Over the past decade, Venezuela has experienced a political and economic crisis that has affected the country’s scientific research development. Currently, the state of HIV research in Venezuela remains unknown. We conducted a systematic review identifying 683 articles over the last 20 years of which only 101 met our inclusion criteria. A decrease in national scientific production was observed starting in 2017, although there was an increase in foreign research on the Venezuelan migrant population. Knowledge gaps were identified in areas such as epidemiology, efficacy and resistance to antiretroviral therapy, and HIV in pregnancy.

## Background

Approximately 92,000 people live with HIV (PLHIV) in Venezuela, with 6,062 new cases and 1,500 deaths reported by 2022 [1]. Since 2016, Venezuela has experienced the highest rate of antiretroviral therapy (ART) interruptions among Latin American countries. The situation worsened in 2017 and 2018 due to limited access to ART, with only 16% of patients receiving treatment by April 2018 [2]. This led to a mass exodus of Venezuelan migrants with HIV, straining the health systems of receiving countries [3–5]. In 2019, nationwide ART access was re-established through a dolutegravir-based regimen, funded by the “Master Plan for Strengthening the Response to HIV, Tuberculosis, and Malaria,” supported by the World Bank [6]. However, the ongoing economic and health crisis in the country has not only impacted care for PLHIV but also hindered research development [7]. Although there was an apparent increase in science and technology funding until 2014 [8], Venezuelan scientific production declined. The loss of 16% of researchers due to migration [9], coupled with decreasing funding, has hampered health research capacity. Due to budget constraints, some state-dependent scientific institutions now operate with private investments [10, 11]. Limited funding poses a significant obstacle for HIV researchers. In response, this study presents a systematic review analyzing original scientific studies on HIV conducted in Venezuela or involving Venezuelan PLHIV. We aim to identify existing research areas, address knowledge gaps, and prioritize future investigations.

## Methods

We conducted a systematic review following the established PRISMA protocol [12]. Our search strategy aimed to extract all potentially relevant studies from PubMed, Scopus, and *Biblioteca Virtual en Salud*. We included articles published between January 1, 2003, and August 20, 2023. The search terms used included DeCS (“Venezuela”, “venezolanos”, “VIH”, and “Sida”) and MeSH (“Venezuela”, “Venezuelan”, “HIV”, and “Aids”) terms. Original studies with specific data related to Venezuelan patients were included. Basic science publications not involving PLHIV samples, commentaries, editorials, narrative or systematic reviews, case reports, and letters to the editor were excluded from the study. The selection process was carried out by two independent reviewers (JAM and MGM), with disagreements resolved by a third reviewer (DAFP). We extracted relevant variables, including the year of publication, study design, sample size, and the region or city where each study was conducted. Based on their main content, each study was grouped into seven categories: clinical behavior, epidemiology, pediatric, migration, treatment, HIV in pregnancy, and miscellaneous.

## Results

**Fig. 1:**
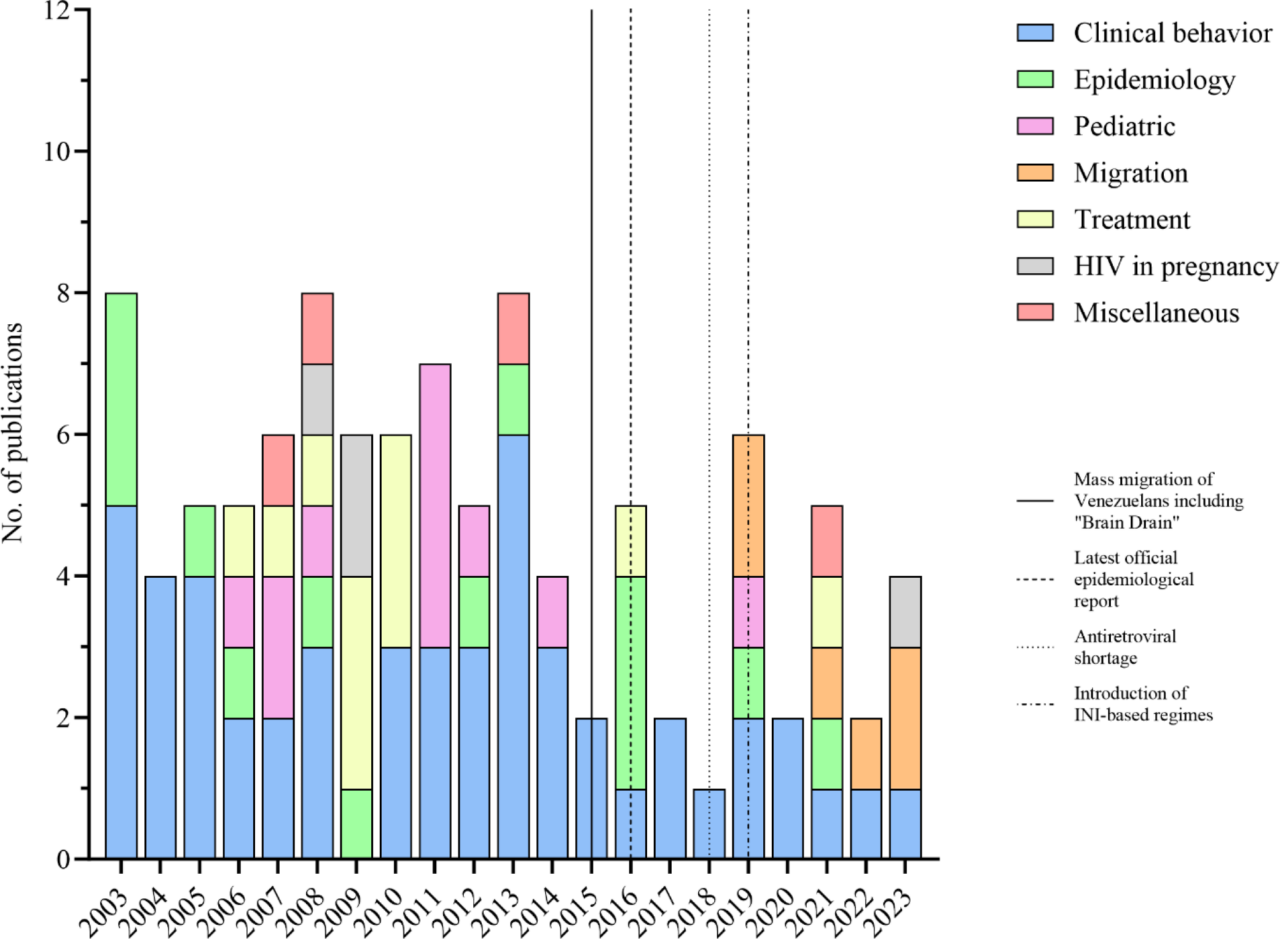
Stacked bar chart on the different types of studies analyzed by year

A total of 928 records were identified in the database, with 683 remaining after the elimination of duplicates. Out of these, 101 studies met the eligibility criteria and were analyzed. Almost all (95%) were observational studies, comprising 90 cross-sectional and six longitudinal studies. Only five studies employed experimental design. The primary focus of research in Venezuela was the clinical behavior of the disease (50%), followed by research on epidemiological aspects (14%), ART (11%), pediatric patients (11%) and migrant PLHIV (6%). Only 4 (4%) studies addressed HIV in pregnancy. A sharp decline in national scientific production was observed in 2017. However, since 2019, scientific production has re-emerged, primarily related to migrant PLHIV (Fig. 1). Out of 101 publications, 11 were produced internationally, 82% of which have been published since 2016. Moreover, since 2019, scientific publications concerning the migrant population have constituted 32% of the total studies conducted up to the date of this inquiry.

## Discussion

This systematic literature review provides insights into the current state of HIV research in Venezuela. While we documented a higher percentage of studies focused on clinical behavior of PLHIV, most were published before 2015. Additionally, gaps exist in research related to prevalence, the efficacy of first-line ART, ART resistance, cardiovascular risk among PLHIV, opportunistic infections, and HIV in pregnancy. Identifying existing knowledge gaps and setting a priority research agenda is crucial for resource allocation and informed health policy.

The decline in clinical research may be attributed to several factors. First, state resources have become scarce, primarily relying on donations or private investments. Additionally, the economic and social deterioration that has affected Venezuela since 2015 has led to the migration of valuable personnel. At least 16% of the research workforce is estimated to have been lost [13]. From 2017 onwards, the decrease in local scientific production is evident; in contrast, a greater production of studies on Venezuelan migrants living with HIV was observed. This increase is attributable to studies conducted mainly in Colombia [4, 14], Peru [5, 15], and Brazil [3, 16], countries that host the largest number of Venezuelan migrants. Furthermore, over the last decade, there has been a dearth of multicenter studies, possibly due to challenges in international relations, hindering access to international funding. Identifying research priorities becomes crucial to allocating limited resources effectively.

We propose the following priority areas for research: (1) current HIV prevalence: examine HIV prevalence in the general population and at-risk groups; (2) efficacy and adherence to ART: conduct multicenter studies involving adult and pediatric populations to assess ART effectiveness and adherence; (3) clinical behavior of HIV infection in Venezuela: focus on patient classification based on disease stage; (4) opportunistic infections and co-infections: explore tuberculosis, cryptococcosis, toxoplasmosis, and *P. jirovecii* infections, estimate the prevalence of co-infection with hepatitis B and C viruses and syphilis; (5) prevalence of ART resistance: particularly examine resistance to first-line regimens with integrase inhibitors; (6) HIV in pregnancy: identify factors associated with late diagnosis of HIV during pregnancy and understand the maternal-fetal outcome. Stimulating basic and clinical scientific research related to HIV is essential. Seeking internal and external funding opportunities will help us understand the current situation and prioritize research needs in line with the Joint United Nations Programme on HIV/AIDS’ 95-95-95 targets.

This study has several limitations that should be considered when interpreting the results. First, we could not obtain the full paper of all articles initially selected for review, so they had to be excluded. Second, we did not have access to other paid search engines, such as Web of Science and Embase, limiting the completeness of the literature search. Also, we did not consider the gray literature, which could have left out the analysis of unpublished studies including undergraduate and graduate theses.

## Conclusions

This review highlights a decline in scientific production since 2017. It also identifies areas of knowledge that have been little or no explored, including epidemiology, ART efficacy, resistance, clinical behavior, and opportunistic infections. Prioritizing research and allocating resources effectively are critical steps. Additionally, we must continue to promote national research while implementing comprehensive care strategies for PLHIV.

## Data Availability

All data and materials in this article are included in the manuscript.

## Abbreviations

HIV: Human immunodeficiency virus
PLHIV: People live with HIV
ART: Antiretroviral therapy
